# Genetically Encoded Voltage Indicators Are Illuminating Subcellular Physiology of the Axon

**DOI:** 10.3389/fncel.2019.00052

**Published:** 2019-03-01

**Authors:** Lauren C. Panzera, Michael B. Hoppa

**Affiliations:** Department of Biological Sciences, Dartmouth College, Hanover, NH, United States

**Keywords:** genetically encoded voltage indicators, axon, synaptic transmission, voltage, action potentials

## Abstract

Everything we see and do is regulated by electrical signals in our nerves and muscle. Ion channels are crucial for sensing and generating electrical signals. Two voltage-dependent conductances, Na^+^ and K^+^, form the bedrock of the electrical impulse in the brain known as the action potential. Several classes of mammalian neurons express combinations of nearly 100 different varieties of these two voltage-dependent channels and their subunits. Not surprisingly, this variability orchestrates a diversity of action potential shapes and firing patterns that have been studied in detail at neural somata. A remarkably understudied phenomena exists in subcellular compartments of the axon, where action potentials initiate synaptic transmission. Ion channel research was catalyzed by the invention of glass electrodes to measure electrical signals in cell membranes, however, progress in the field of neurobiology has been stymied by the fact that most axons in the mammalian CNS are far too small and delicate for measuring ion channel function with electrodes. These quantitative measurements of membrane voltage can be achieved within the axon using light. A revolution of optical voltage sensors has enabled exploring important questions of how ion channels regulate axon physiology and synaptic transmission. In this review we will consider advantages and disadvantages of different fluorescent voltage indicators and discuss particularly relevant questions that these indicators can elucidate for understanding the crucial relationship between action potentials and synaptic transmission.

## Introduction

### The AP Is a Flexible Currency of Cellular Communication

The linkage between electricity and signaling of the nervous system was first postulated by Galvani in 1791 after observing musculature contraction in the frog by applying electrical discharges directly onto nerve fibers (Ashcroft, 2012). It was not until the 1950s that Hodgkin and Huxley made the fateful recordings of membrane potential in the squid giant axon to discover voltage-gated ion channels give rise to propagating electrical signals known as action potentials (APs) (Hodgkin et al., 1952; Hodgkin and Huxley, 1952). The AP is the informal currency of information transfer between nerves that is enabled by the precisely timed flow of positively charged ions for both the depolarization (Na^+^ influx) and repolarization (K^+^ efflux) phases of the waveform. The invention of the patch-clamp enabled recordings of the AP from several small neurons across the nervous system of several species. Although the squid axon had a binary firing response to different stimulations, several firing patterns were discovered that differed considerably between cell types (Llinas, 1988; Bean, 2007). This diversity includes the fast spiking inhibitory parvalbumin and Purkinje neurons compared to the slower firing of excitatory pyramidal neurons of the hippocampus and dopaminergic neurons in the substantia nigra (Grace and Bunney, 1984; McCormick et al., 1985; Connors and Gutnick, 1990; Raman and Bean, 1999; Brown et al., 2009; Gentet et al., 2010). Many of these differences in firing rates arise from different combinations of Na^+^ and K^+^ channels (Covarrubias et al., 1991; Dodson et al., 2002; Dodson and Forsythe, 2004; Engel and Jonas, 2005; Jan and Jan, 2012) which also give rise to different shapes of AP waveforms. APs in several fast firing neurons such as Purkinje neurons are characterized as having very narrow widths (∼180 μs) and fast rise times, while those of some slower firing neurons such as CA1 pyramidal neurons are much broader (∼810 μs) (Bean, 2007). This establishes the waveform as a plastic signal that encodes potentially interesting neurobiological information and rests upon the specific combinations of ion channels expressed at the cellular level. However, how different localization of channels within cells can modulate the shape of the AP at the subcellular scale is less well understood due to technical limitations of electrophysiology.

### The AP Waveform at Presynaptic Terminals Is Not Equivalent to the Somatic Waveform

After initiation in the axon initial segment adjacent to the soma, an AP rapidly propagates along the axon until it reaches the presynaptic terminals (Kole and Stuart, 2012). These terminals transduce incoming electrical signals (APs) into chemical signals (neurotransmitter release). Changes in transduction efficiency are the basis of memory formation, and chronic weakening of synaptic transduction is associated with pathologies such as Alzheimer’s. The coordination of Ca^2+^ entry that results in vesicle fusion is directed by the AP. The depolarizing event triggers the opening of voltage-gated Ca^2+^ channels (Cavs) in a presynaptic terminal and subsequent vesicle fusion (Dodge and Rahamimoff, 1967; Llinas et al., 1981; Rosenmund et al., 1993; Schneggenburger and Neher, 2000). The AP is a command signal that sharply controls the open probability and open duration of Cavs and thus tightly regulates the synaptic concentration of Ca^2+^ (Llinas et al., 1981; Schneggenburger and Neher, 2000; Lisman et al., 2007). Vesicle fusion is a supra-linear process that is steeply dependent (by a 3rd order power-law) on Ca^2+^ entry at the synapse (Dodge and Rahamimoff, 1967; Augustine et al., 1985; Zucker and Fogelson, 1986; Schneggenburger and Neher, 2000). Thus, the AP is well positioned for modulating synaptic function, and small changes in the AP waveform have been shown to exert enormous impact on synaptic transmission (Sabatini and Regehr, 1997; Borst and Sakmann, 1999; Bischofberger et al., 2002; Rama et al., 2015).

An unexpected gap in the well-studied field of synaptic transmission is the measurement of the presynaptic AP waveform in small *en passant* synapses that dominate the central nervous system. Somatic recordings are generally not an accurate proxy for activity at nerve terminals, though the comparisons have been hard to come by using classic electrophysiology. The first detailed subcellular comparison of the AP waveform occurred in granule neurons from the dentate gyrus and their mossy fiber boutons which are large enough to allow electrical access (>3 μm). These recordings demonstrated a dramatically narrower AP at the mossy fiber terminal and provided the first physiological evidence that these two cellular compartments are quite independent with physiological consequences for synaptic transmission (Geiger and Jonas, 2000). More recently it was found that the nerve terminals of Purkinje neurons (∼3 μm) also have very different waveforms due to a unique balance of Na^+^ and K^+^ channels compared to the cell body and, more surprisingly, compared with adjacent axon (Kawaguchi and Sakaba, 2015). However, outside of these examples, whole-cell patch clamp is not technically feasible for decoding AP waveforms and molecular modulators in *en passant* synapses due to their sub-micron diameters (Shepherd and Harris, 1998; Mishchenko et al., 2010). Recordings from the few other large and accessible mammalian terminals has further demonstrated different AP waveform shapes when recorded from pituitary nerves (Jackson et al., 1991) and the calyx of Held (Sierksma and Borst, 2017). This divergence makes understanding the role of the AP in nerve terminals of even higher interest, especially given the host of recent channelopathies attributed to the presynaptic terminal and axon (Kullmann, 2010; Child and Benarroch, 2014; Vivekananda et al., 2017).

The development of optogenetic indicators other than voltage has provided new opportunities for imaging neural activity within the small *en passant* synapses of the central nervous system. The rapid advancements for improving genetically encoded Ca^2+^ indicators (Chen et al., 2013) has revolutionized recording physiology with large signal-to-noise ratios (SNRs) that enable their use in defined compartments or cell types *in vitro* and *in vivo* (Sofroniew et al., 2016). This review will highlight the recent advances in voltage imaging specifically for the use of resolving APs within the axon and synaptic terminals. We will go on to highlight areas where optical measurements of voltage have recently been deployed to contribute to new knowledge of axon and synaptic physiology, as well as potentially interesting future directions.

### Ideal Properties of Genetically Encoded Voltage Indicators

Experimental use of genetically encoded voltage indicators (GEVIs) to record physiological voltage changes in the axon is still hindered at this time by low signal-to-noise ratios. While this is a consideration in all cells, it is particularly difficult for resolving the AP in small diameter (<300 nm) structures such as the axon that emit few photons due to limited surface area. This is made more difficult for recording transient APs that are fully resolved in 1–3 ms. Thus, photons emitted by fluorophores in response to voltage changes must be collected in very brief acquisition windows (<1 ms), making brightness paramount for accurate detection of the AP. It is also much easier to observe signals with a larger percentage change in fluorescence (ΔF/F) above any optical noise unrelated to your signal. We have reported the sensitivity and relative brightness of several recent iterations of reported GEVIs (Table 1). A second desired attribute for a GEVI, especially in the context of resolving an AP waveform, is that the indicator would have changes in fluorescence or kinetics for the depolarization and repolarization phases with taus on the order of 100 μs. Minimally, taus of fluorescent change need to be <1 ms for accurate detection of AP firing rates at 100 Hz; tau constants for both phases are also reported (Table 1). Final key considerations of GEVIs when combining with other optogenetic or fluorescent proteins, as well as determining potential use with 2-photon excitation, are their excitation and emission spectra. We have color-coded indicators in Table 1 for clarity based on the single photon excitation energy/color (blue, green, and red) and divided by mechanism of fluorescence as detailed below.

**Table 1:** Comparison of contemporary GEVI characteristics.

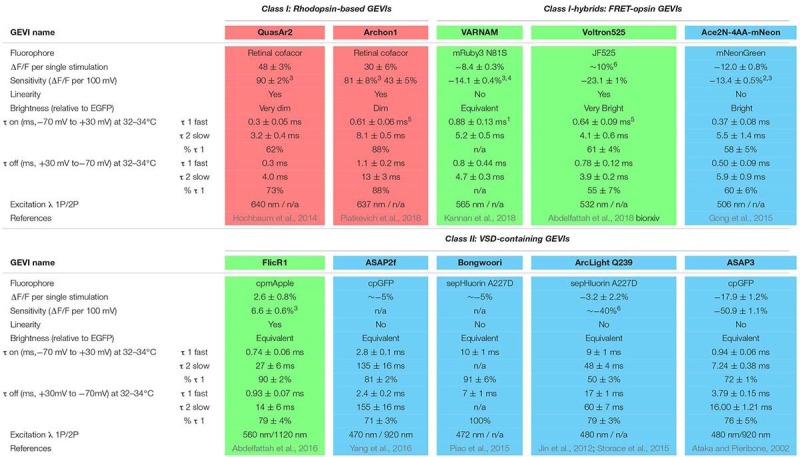

*GEVIs are grouped according to their voltage-sensing mechanism and color-coded according to their single photon excitation wavelength (blue, green, red). Superscripts denote the following: (1) experiments to determine VARNAM kinetics performed at room temperature. (2) Experiments to determine sensitivity of Ace-mNeon published in Kannan et al. (2018) from a 120 mV depolarization. (3) Measured in HEK cells. (4) In response to a 120 mV depolarization. (5) Experiments performed at unknown temperature. (6) Value estimated from figure in cited paper.*

### Voltage-Sensitive Dyes

Voltage-sensitive (VS) dyes already have many ideal characteristics for voltage-indicators: they are bright, photostable and have very fast kinetics (Zhou et al., 2007; Huang et al., 2015; Woodford et al., 2015), although many VS dyes alter properties of the membrane itself (Peterka et al., 2011). That important consideration aside, perhaps the most significant limitation of voltage-sensitive dyes is their lack of specificity. Lipophilic dyes stain all cell membranes when loaded extracellularly, so voltage-dependent fluorescence signals cannot be distinguished between cell types. Intracellular loading through glass electrodes gives excellent specificity and has been useful to studying axon physiology, but must be loaded cumbersomely through electrodes and allowed to diffuse through the cell, limiting their use *in vivo* or for measuring multiple adjacent cells.

### GEVIs in the Axon

There is a rich abundance of fluorescent voltage indicators available with different characteristics of speed, color, brightness, and sensitivity. When assessing indicators, each must be weighed for its inherent strengths and weaknesses in consideration of the research question being asked. Given the diversity of applications for voltage imaging and an explosion of engineered GEVIs, many reviews have been published comparing GEVIs and their applications (Emiliani et al., 2015; Knopfel et al., 2015; St-Pierre et al., 2015; Storace et al., 2016). Here we will focus solely on the current toolkit of GEVIs available as of this publication that are best suited for investigating voltage changes in the axon, with a focus on detecting and resolving AP waveforms. These GEVIs have historically fallen into two main classes: microbial rhodopsin-based indicators, and voltage-sensing domains (VSDs) of phosphatases fused to fluorescent proteins. We will follow the naming style of previous GEVI discussion (Nakajima et al., 2016) and label these two types Class I and Class II, respectively, and start our discussion with a brief timeline of GEVI development beginning with Class II probes. A final group of indicators currently in development, Class I-hybrids, use Förster resonance energy transfer between a microbial rhodopsin and a fluorescent protein to report changes in voltage (Gong et al., 2014).

## Comparison of GEVIs

### Class II: VSD-Containing GEVIs

The earliest VSD GEVIs engineered contained voltage-sensing domains of naturally occurring ion channels fused with fluorescent proteins (Siegel and Isacoff, 1997; Ataka and Pieribone, 2002). Unfortunately, these initial probes suffered from limited plasma membrane expression and poor targeting which hindered their use in mammalian systems (Storace et al., 2016). Membrane expression was improved by replacing the voltage sensor of voltage-gated K^+^ channels (Kvs) with a domain of the *Ciona intestinalis* voltage-sensitive phosphatase (Ci-VSP) (Murata et al., 2005).

There are two strategies used by Class II indicators to alter fluorescence. Probes including ArcLight (Jin et al., 2012) and an ArcLight derivative, Bongwoori (Lee et al., 2017), feature Ci-VSP fused to an intracellular super ecliptic pHluorin. Membrane depolarization induces a conformational change in the VSP which alters fluorescent emission. This approach yields reporters that are very bright yet also relatively slow, which makes them more suitable for detecting small changes such as graded potentials as opposed to APs. A second strategy for changing fluorescence takes advantage of an innovation in the engineering of fluorophores with the development of circularly permuted fluorescent proteins (cpFPs). The introduction of new termini into the tertiary structure of a fluorophore generates space for the fusion of a voltage sensing domain; conformational changes to the permuted protein alter fluorescence (Baird et al., 1999). ASAP1, a fusion construct of an extracellular cpGFP to the VSP of *Gallus gallus* (St-Pierre et al., 2014), improved upon the speed and sensitivity of previous VSP sensors including ArcLight. An ASAP variant, ASAP2f, demonstrated robust fluorescence changes ∼14% larger than ASAP1 in response to single stimuli (Yang et al., 2016); the current iteration, ASAP3, has sensitivity and speed enough to detect both subthreshold events and individual spiking up to 100 Hz in acute brain slices (Chavarha et al., 2018). A red-shifted variant, FlicR1, was generated by fusing a Ci-VSP domain to cpRFP (cpmApple) (Abdelfattah et al., 2016). One disadvantage of the previously mentioned GFP-based reporters is that the conformational changes that lead to photon emission suffer from slow kinetics (τ_fast_> 3 ms) that limit the accurate resolution of AP waveform dynamics. Notably, however, they have been engineered to have very high brightness which facilitates detection of both APs and subthreshold deflections.

### Class I: Rhodopsin-Based Indicators

The discovery of a light-driven proteorhodopsin found in marine bacteria (Beja et al., 2001) led to the development of an entirely new branch of GEVIs that uses the voltage-dependent protonation of a rhodopsin chromophore, retinal, to generate fluorescence. In the wild, light drives protons through the proteorhodopsin, resulting in a change in emission spectra of the protein. Mutagenesis eliminated light-induced proton pumping (Dioumaev et al., 2003) to yield a proteorhodopsin optical proton sensor (PROPS) that revealed electrical spiking up to 1 Hz in *Escherichia coli* (Kralj et al., 2011b). Membrane localization of PROPS in eukaryotic cells failed, so the principle of reversing pump direction was applied to Archaerhodopsin 3 (Arch) from *Halorubrum sodomense*, which had known eukaryotic plasma membrane targeting (Kralj et al., 2011b). Site-directed mutagenesis of Arch yielded QuasArs (‘quality superior to Arch’), which retained the rapid submillisecond kinetics of the original Arch voltage indicators but demonstrated much improved sensitivity and linear fluorescence responses to voltage changes (Hochbaum et al., 2014). The opsin core of QuasAr2 was enhanced to develop the Archon molecules (Piatkevich et al., 2018); compared to earlier Arch-derived sensors, Archon1 exhibits increased brightness, with high enough sensitivity to detect subthreshold voltage events as small as ∼5 mV with a SNR of 2 or greater and only a small decrease in speed compared to the original QuasAr2 GEVI (Piatkevich et al., 2018).

One significant limitation of microbial rhodopsin GEVIs is their low quantum efficiency which makes them at least 30 times dimmer than GFP (Hochbaum et al., 2014). Low photon release demands trial averaging to resolve AP waveforms and makes high frequency spike detection difficult. Dim fluorescence also necessitates increased laser power and risk of photobleaching and phototoxicity, however, this latter concern is minimized with red-shifted indicators due to the relative lack of autofluorescence in this spectrum. A significant advantage over Class II GEVIs is their fast kinetics and large dynamic ranges that enable resolution of individual AP waveforms even at high frequency firing rates akin to electrophysiological recordings.

### Class I-Hybrids: FRET-Opsin Indicators

To facilitate axonal AP recording, an ideal GEVI would combine the best characteristics of both classes: the speed of rhodopsin indicators with the brightness of VSD-containing probes. FRET-opsin hybrid reporters use bright fluorophores or synthetic dyes as donor molecules and rapid opsins as acceptor molecules, resulting in electrochromic FRET (eFRET). A promising recent hybrid probe fused a rhodopsin variant from *Acetabularia acetabulum* (Ace2N) with mNeonGreen (Gong et al., 2015). The resultant indicator has improved fluorophore brightness and fast (∼1 ms) kinetics with a more linear response to voltage than its predecessor (Gong et al., 2014). Ace2N-mNeon not only can resolve APs in single cells within live animals, but provides robust responses *in vivo* with resolution for single cell and single AP recording in mice and fruit flies (Gong et al., 2015). An exciting red-shifted indicator with comparable characteristics, VARNAM, couples Ace with mRuby3 (Kannan et al., 2018).

A variation of the FRET-opsin strategy is Voltron, which replaces the fluorescent protein FRET donor with a synthetic fluorescent dye. Dyes can be chosen based on their excitation and emission spectra and generally exhibit far superior optical properties of brightness or photostability. Voltron enables specific localization of the synthetic dyes by utilizing a self-labeling protein tag domain that covalently binds the donor dye and permanently couples it to an Ace2N voltage-sensing domain. Voltron bound to the dye molecule JF_525_ (Grimm et al., 2017) (Voltron_525_) exhibited the greatest sensitivity (-23 ± 1% per 100 mV) of FRET-opsins (Abdelfattah et al., 2018).

Hybrid FRET-opsin indicators couple the superior brightness of VSD-containing GEVIs with speed approaching opsin-based GEVIs (∼1 ms). There are two significant disadvantages to FRET probes. First is a reduction in their sensitivity to voltage and curtailed response ranges. The second is that they use up much more of the excitation-emission spectrum that limits their combined expression with other genetically encoded indicators or actuators. The newest versions of these probes are bright enough for AP detection, but it will likely be challenging to resolve AP waveforms with their current kinetic properties in most nerve cells.

## Optical Approaches to Probe Molecular Control of Presynaptic APs

Bulk-loaded VS dyes have been successfully deployed to explore presynaptic APs from collections of fine terminals and axons emanating from parallel fibers of granule cells in the cerebellum (Sabatini and Regehr, 1997, 1999). These critical measurements established the impact that altering AP width has on Ca^2+^ entry and neurotransmitter release in small nerve terminals that followed previous early electrophysiology in invertebrates (Byrne and Kandel, 1996). However, this technique of extracellular dye labeling is too non-specific to measure axonal responses as measurements are confounded by surrounding tissue including astrocytes, dendrites and heterogeneous axonal populations. The development of brighter VS dyes with fast kinetics (Yan et al., 2012) permitted single cell recording with intracellular loading. The “blue dye” type of VS dye (Zhou et al., 2007) was used to great effect to study axon collaterals and the cell bodies of layer 5 pyramidal cells, and pharmacological experiments demonstrated that narrow APs, as discussed above, are the result of a high density of Kv1 channels (Foust et al., 2011). 2-photon recording of hemicyanine VS dyes loaded into inhibitory stellate cells of the cerebellum revealed that Kv3 channels dominate the repolarization of fast APs in the presynaptic terminals while Kv1 channels were influential at the initial segment (Rowan et al., 2014). Adding to this diversity of K^+^ channels modulating presynaptic APs are recordings from inhibitory neurons in the neocortex that identified different combinations of BK and Kv1 channels in fast-spiking and somatostatin-expressing interneurons (Casale et al., 2015).

The engineering of GEVIs with fast kinetics (Kralj et al., 2011a) has also enabled detailed measurements of the AP waveform in single cells with trial averaging. The first study to utilize the rhodopsin indicator Arch for measuring the presynaptic AP in *en passant* terminals leveraged its linear readout to calibrate the responses by forcing the membrane potential close to 0 mV with the cation-selective ionophore gramicidin. Excitatory hippocampal terminals were found to be rich in Kv1 channels and invaded by APs with low (+7 mV) overshoots that are ideally positioned for modulating Cav open probability (Hoppa et al., 2014). Arch was also used to robustly identify a dominant role for BK (Slo) channels at the *Drosophila* neuromuscular junction as a novel regulator of synaptic transmission (Ford and Davis, 2014). Thus, both VS dyes and GEVIs have already begun contributing to our understanding of ion channel distributions in a variety of synaptic terminals.

### The Use of Optical Voltage Measurements to Study Compartmentalized Control and Temporal Aspects of the AP in the Axon

A recent review has highlighted various aspects of axon physiology to control the propagation of APs and compartmentalized signaling domains within the axon (Rama et al., 2018). Here, we discuss how VS dyes and GEVIs have contributed to our understanding of electrical signaling in the axon. The recent use of VS dyes has revealed remarkable levels of heterogeneity in the shape of the AP waveform between individual synapses of stellate neurons (Rowan et al., 2016). This paper convincingly introduces the AP as a plastic signal that modulates synaptic strength. *In vivo* recordings of voltage across the axonal arbors of Mi1 cells in the *Drosophila* visual system revealed incredible diversity between the AP waveforms that invaded presynaptic terminals depending on which cellular layer they innervated (Yang et al., 2016). These results were strongly correlated with differences in Ca^2+^ imaging, indicative of an important role of the AP waveform in sensory physiology. Mechanisms underlying this heterogeneity are still being resolved. Toward this end, measurements of AP heterogeneity along the axon of excitatory hippocampal neurons *in vitro* revealed a novel role for Navβ2 subunits in enabling successful propagation across axonal branch points (Cho et al., 2017). Thus, non-invasive multi-point recording along an axon is a distinct advantage of non-invasive optical voltage measurements. GEVIs have also contributed to our understanding of synaptic plasticity and how AP shape alterations during high frequency firing can depress or facilitate synaptic transmission (Jackman and Regehr, 2017). A recent publication was able to demonstrate *ex vivo* recordings of axons using GEVIs to explore AP broadening in the fine axons of mossy fibers (Ma et al., 2017), reminiscent of original recordings from terminals (Geiger and Jonas, 2000).

### Future Directions of GEVIs to Explore Synaptic Transmission

**FIGURE 1:**
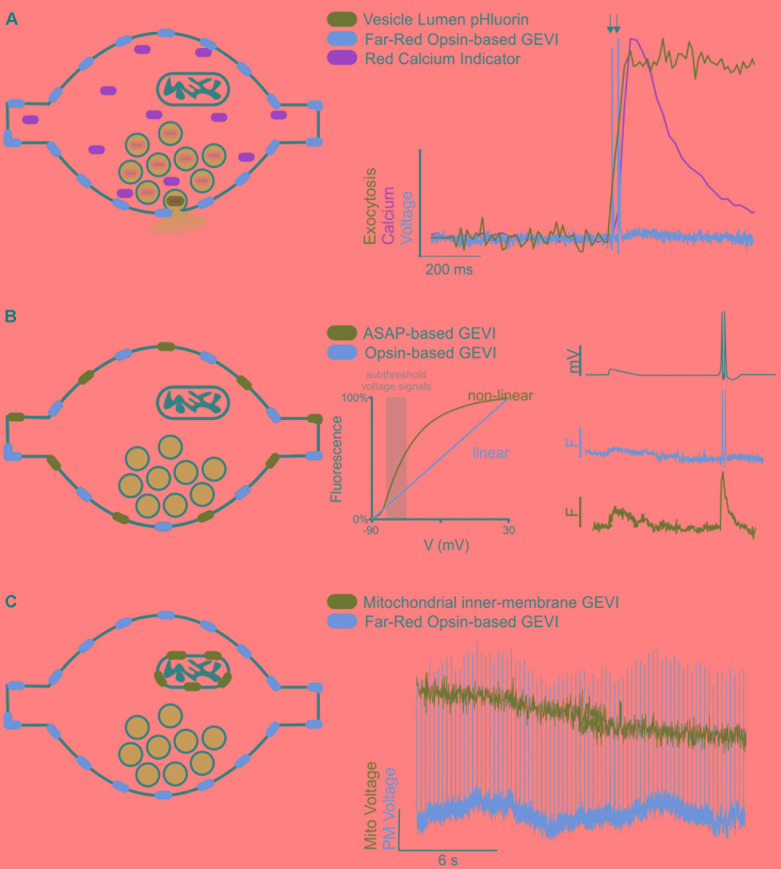
Future directions of GEVIs to explore synaptic transmission. **(A)** Cartoon of a synapse expressing a far-red rhodopsin-based GEVI on the membrane and a cytosolic red Ca^2+^ indicator. Vesicles (blue circles) contain pHluorin, a vesicular lumen-targeted pH-sensitive reporter of exocytosis (*left*). Theoretical traces (*right*) demonstrating recording of two single AP stimulations (arrows) superimposed with theoretical fluorescence traces of the Ca^2+^ and exocytosis response in the same cell. **(B)** Cartoon of a synapse expressing two spectrally separate, membrane-targeted GEVIs (*left*). Subthreshold events (gray box, *center*) are in a voltage range better detected with large fluorescence changes by a non-linear indicator than a linear indicator. A theoretical voltage trace (*right, top*) of a subthreshold event followed by two single AP stimulations. Theoretical fluorescence traces of the linear (*middle*) and non-linear (*bottom*) indicators demonstrate how combining indicators could help resolve both sub- and suprathreshold events in the same cell. **(C)** Cartoon of a synapse expressing a membrane-targeted far-red GEVI, and a mitochondrial-targeted green GEVI (*left*). Theoretical fluorescence traces of both indicators demonstrate simultaneous recording of mitochondrial and membrane voltage dynamics in the same cell.

A recent review of voltage indicators predicted that the future of voltage indicators, especially regarding their use *in vivo*, is brighter, more sensitive, and redder; we would hasten to add faster as well for use in resolving an AP (Xu et al., 2017). Indeed, for many aspects of signal detection the use of red excitation and far-red emission GEVIs and VS dyes has distinct advantages as previously summarized (Xu et al., 2017). However, a host of fluorescent green (blue light excited) dyes and genetically encoded indicators exist to measure other properties of synaptic transmission including vesicle lumen pH, calcium, and neurotransmitters themselves. Therefore, thanks to the large spectral separation of far-red VS dyes and GEVIs from GFP-based reporters, future experiments can combine these green emission indicators with red voltage sensors to monitor not only how ion channels and their binding partners modulate voltage but how that translates to other aspects of synaptic function (Figure 1A). This spectral separation has already been combined with blue-light excited ion channels to investigate cellular (Emiliani et al., 2015) and subcellular aspects of neural excitation (Fan et al., 2018).

Multiple GEVIs with divergent spectral emission also be combined with great effect to study interesting aspects of axon physiology beyond the AP, especially if the two indicators have divergent sensitivities (linear and non-linear) to voltage. This situation could be exploited in future studies to detect different types of voltage signaling in the axon (Figure 1B). APs are difficult signals to measure due to their very rapid kinetics, but easy in terms of the large (>80 mV) voltage change, thus fast and linear indicators are optimal. However, there are also several types of subthreshold signals within the axon that do not initiate APs but do influence synaptic transmission that are slower and have smaller voltage changes (<10 mV) (Zbili et al., 2016; Rowan and Christie, 2017). Currently, small voltage changes in the axon are more challenging to detect using linear indicators with rapid kinetics such as an opsin-based GEVI including QuasAr, but less so for slower, brighter GEVIs that have non-linear responses to voltage such as ASAP (St-Pierre et al., 2014). These two indicators could be combined and simultaneously monitored to explore sub-threshold voltage in relation to AP propagation and modulation of waveform kinetics.

A third area of future interest measuring voltage with GEVIs would be to move beyond the plasma membrane. Given that light is not constrained by any size restriction, we can move much smaller than the synaptic terminal and explore how voltage might control other aspects of synaptic transmission at the level of organelles (Figure 1C). In theory, any membrane can use voltage for signaling or function including, but not limited to, synaptic vesicles and mitochondria. Voltage modulation in organelles is severely understudied and could benefit from adapting GEVIs to new forms of subcellular targeting such as those recently deployed for targeting Archaerhodopsins to synaptic vesicles and lysosomes (Rost et al., 2015). We are in the infancy of voltage detection with light and the future seems bright to learning new aspects of neurobiology in health and disease states.

## Author Contributions

LP and MH conceptualized and designed the study, and drafted and revised the article.

## Conflict of Interest Statement

The authors declare that the research was conducted in the absence of any commercial or financial relationships that could be construed as a potential conflict of interest.
